# Localization effect on AMS fabric revealed by microstructural evidence across small-scale shear zone in marble

**DOI:** 10.1038/s41598-019-53794-y

**Published:** 2019-11-25

**Authors:** V. K. Kusbach, M. Machek, Z. Roxerová, M. Racek, P. F. Silva

**Affiliations:** 1 Institute of Geophysics AS CR, v.v.i, Boční II/1401, 14131 Prague 4, Czech Republic; 2 Charles University Prague, Institute of Petrology and Structural Geology, Prague 2, Czech Republic; 3 Insituto Superior de Engenharia de Lisboa (ISEL, IPL), Physics Department, Lisbon, Portugal; 4 Instituto Dom Luiz (IDL - Univ. Lisboa), Lisbon, Portugal

**Keywords:** Geodynamics, Structural geology, Geomagnetism, Geomagnetism, Geophysics

## Abstract

Anisotropy of magnetic susceptibility (AMS) is regularly applied as a tool to infer structural analysis of deformation and flow in rocks, particularly, with low anisotropy. AMS integrates the magnetic signature of crystallographic and shape preferred orientation of all mineral grains present in the rock microstructure. Those preferred orientations result from multiple processes affecting the rock during its evolution, therefore the desirable AMS-strain relationship is not straightforward. Here we show that due to localization of deformation, AMS is indirectly dependent on the magnitude and character of deformation. In order to decipher the AMS-strain relationship, AMS studies should be accompanied by microstructural analyses combined with numerical modelling of magnetic fabric. A small-scale shear zone produced by single deformation event was studied. The resultant AMS fabric is “inverse” due to the presence of Fe-dolomite and controlled by calcite and dolomite crystallographic preferred orientations. The localized deformation resulted in the angular deviation between macroscopic and magnetic fabric in the shear zone, systematically increasing with increasing strain. This is a result of the presence of microstructural subfabrics of coarse porphyroclasts and fine-grained recrystallized matrix produced by localization.The localization of deformation is a multiscale and widespread process that should be considered whenever interpreting AMS in deformed rocks and regions.

## Introduction

AMS as a dimensionless material parameter indicates the degree of magnetization of a sample in response to an applied magnetic field in different directions. AMS measurements reflect an integrated magnetic signal of crystallographic and shape preferred orientation (CPO and SPO) of all mineral grains forming the rock; and traditionally have been used as an effective tool to decipher mineral fabric in various rock types. However, rock microstructures are a consequence of superpositions of a wide variety of processes, such as, sedimentation, diagenesis, magma flow, deformation and metamorphism, among others. These processes give origin to individual subfabrics, which, form the microstructure during rock evolution^1^. Consequently the microstructure of a deformed rock is often a complex combination of distinct microstructural features of diverse orientations and strengths. Therefore, it is not only important to evaluate the contributions of the main susceptibility carriers to the anisotropy^2^, it is also important to identify the processes responsible for AMS development and their eventual superposition^3–13^.

AMS has been often used as a technique of structural analysis documenting the orientation and magnitude of individual deformation events. The AMS ellipsoid has a similar representation to a finite strain ellipsoid, with the principal axes having a reasonably straightforward structural significance with the exception of certain AMS carrier mineral phases^2^ like siderite and other carbonates of high Fe content and amphibole as well as single domain magnetite. It may be used as a proxy to determine the strain in rocks and the AMS-strain relationship has been studied both in experimental and in natural systems^14–17^. The similarity between the magnetic and strain ellipsoids has been well-documented for their orientations^18–21^, however, their magnitudes are more complexly related to lithology^17,21,22^, mineral abundances^15,23^ and rock strain memory^6,24^.

Studies of AMS-strain correlation have traditionally taken one of two approaches. First numerical modelling that simulates magnetic fabric development^25–29^ and second empirical correlations between strain markers and the susceptibility ellipsoid^26^ in analogue models and natural samples. Many natural cases studies have shown that there exists a positive correlation between the degree of anisotropy P_j_ and strain as long as the magnetic mineralogy is constant^30,31^. Additionally, the significance of the primary fabric has been noted in experimental studies^24,32–34^.

In the present study, which describes the evolution of the AMS-strain relationship in a marble hosted small-scale shear-zone, we follow the still valid and often overlooked suggestion made by Borradaile^24^: *“Magnetic fabrics should not be used for routine methods of “strain analysis” without further study. A more profitable avenue is to determine how the susceptibility and strain tensors relate, taking account of the actual strain-response model (deformation mechanisms), and the actual mineralogical sources of susceptibility in that rock, including phases produced by metamorphism. A fruitful approach would be to consider the tensorial combination of coexisting magnetic subfabrics and successive episodes of magnetic fabric development along the lines initiated by Hrouda*^35^*.“* Our goal is to provide a detailed microstructural and rock magnetic study to uncover how AMS is controlled by microstructure formed during a single deformation event. In this manuscript, AMS, microstructural and textural changes are documented along two profiles in a calcite shear zone and the response of AMS to microstructural evolution under a deformational gradient is evaluated. The aim is to bring new insights about the ability of joint microstructural and AMS analysis to describe the deformational evolution.

## Geological and Structural Setting

**Figure 1:**
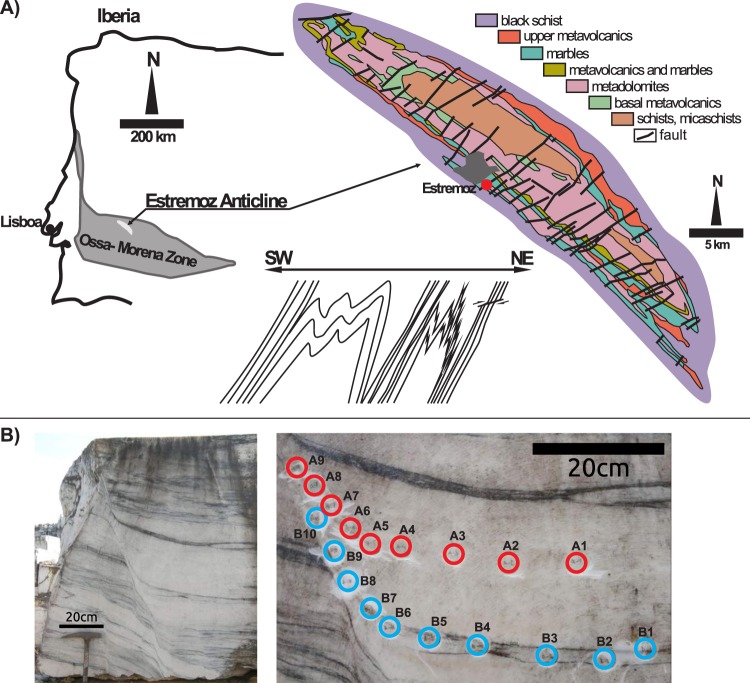
Simplified geological map. (**A**) Regional geological map with a schematic picture of the structural record in the area. (**B**) Field photograph of the studied shear zone in marble characterised by alternating bands, with the position of the oriented samples marked.

Exceptional outcrops of deformed marble are exposed in the SW limb of the Estremoz Anticline in Portugal (Fig. 1A), which is part of the Volcanic–Sedimentary Complex of the Estremoz of upper Cambrian age^36^. This anticline corresponds to a major Variscan structure of the Ossa-Morena Zone that resulted from two Variscan folding phases under greenschist metamorphic conditions. The marble is characterised by coarse-grained foliation parallel to alternating bands of white, almost pure calcite up to 30 cm thick, and thinner grey bands (Fig. 1B).

The deformation history of the area of Estremoz is dominated by pervasive isoclinal folding resulting in steep SW dipping axial plane cleavage (Fig. 1A). The studied small-scale shear zone (SZ) was sampled on a marble block in an abandoned quarry WSW of Estremoz (38°50′09.0″N 7°34′01.3″W, SW Portugal). Based on the observed structural superposition, the studied SZ (Fig. 1B) is one of the subhorizontal mostly brittle to ductile shear zones crosscutting the SW-dipping axial plane cleavage (Fig. 1A). In the SZ, foliation is continuously curved towards the centre where most of the strain is localized in a fine-grained shear plane (Fig. 1B). Two structural profiles across this SZ were studied and sampled. Profile A is located in the pure calcite rocks (Fig. 1B, nine samples) and profile B in the grey zone enriched by accessory minerals (Fig. 1B, ten samples).

## Results

### Estimated strain

The studied SZ belongs to the class of ductile shear zones where no macroscopic fracture is involved^37^ and showing a continuous displacement gradient. The SZ results from simple shear deformation since markers of non-simple shear are missing. Displacement (~41.5 cm) on the SZ is approximately 3 times the width (~13 cm).

**Figure 2:**
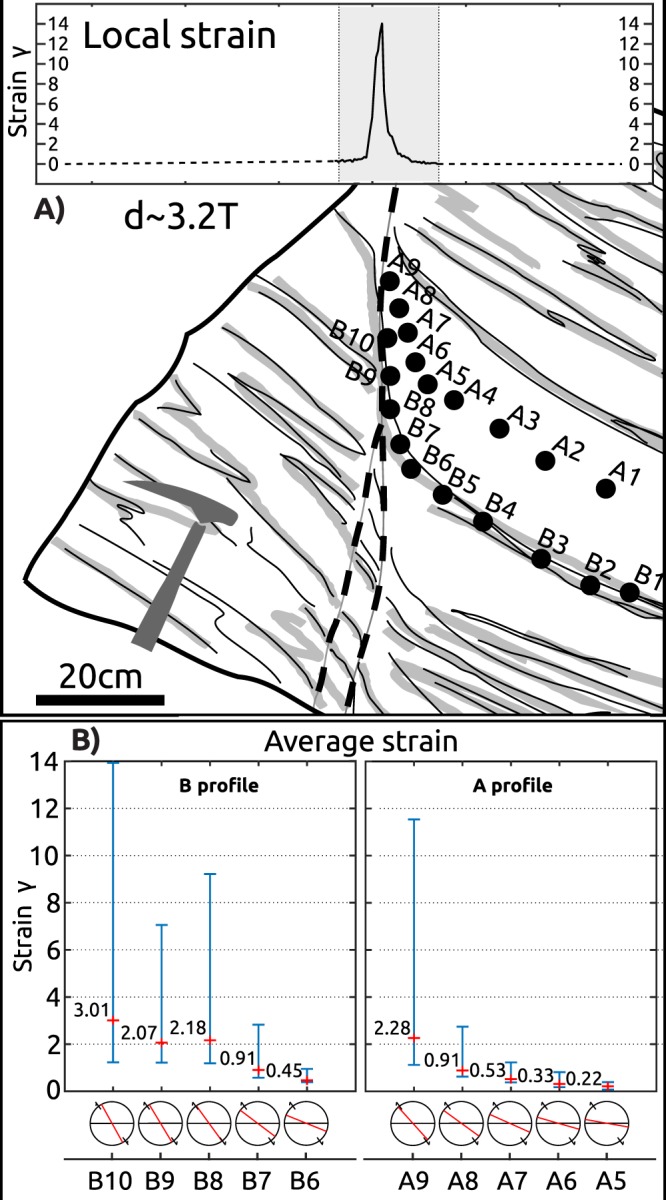
Strain analysis for studied shear zone and individual samples. (**A**) Simplified sketch with position of studied sample profiles and individual oriented samples with respect to shear strain profile across shear zone. Shear strain is calculated from linear feature/foliation re-orientation with a 3 mm resolution from detailed photography, assuming only simple shear. (**B**) Diagram representing average strain on the macroscopic scale of AMS calculated for individual cylindrical samples in both profiles. Average strain (the local incremental strain is integrated over sample volume), minimal and maximal local strain within each sample is visualised. At the bottom is shown orientation of the macroscopic fabric (red line) with respect to the orientation of the primary fabric (black line) for each sample.

The shear strain (γ) profile is estimated from linear feature/foliation re-orientation of passive markers^38^ with a 3 mm space resolution from detailed photography, assuming only simple shear. The small step produces local strain values of γ_local_~14 in the centre of the SZ (Fig. 2A). To obtain an average strain for individual AMS samples, the strain profile was integrated over their respective volumes (Fig. 2B). The maximal average strain in the SZ core is identified in sample B10 (γ_average_ ~3, γ_local_ ~14; Fig. 2B).

### Petrography

The marble white bands are dominated by almost pure calcite. The thinner grey bands are contaminated mainly by quartz, dolomite, muscovite and kaolinized muscovite, phlogopite, Mg chlorite, apatite, tourmaline, K-feldspar and zircon as determined by SEM-EDS analyses. X-ray diffraction of the pure calcite white bands revealed only trace amounts of mica and up to 0.3% of quartz and other phases, while the grey bands contain up to 5.8% of quartz, 2% of dolomite and 0.3% of mica.

The chemical composition of calcite, dolomite and muscovite were analysed both external to the SZ (B5) and within the SZ core (B10) by SEM-EDS. Outside the SZ, the calcite is characterised by MgO = 0.48–1.40 wt% and FeO = 0–0.35 wt%, dolomite by MgO = 18.07–18.46 wt% and FeO = 1.78–2.23 wt% and muscovite by FeO = 2.45–4.09 wt% (Supplementary Table S1). Internally the SZ main mineral phases show lower impurity contents (Supplementary Table S1). The chemical composition maps (Supplementary Fig. S1) show concentrations of Fe within tiny stripes rich in secondary phases and of Mg in the indistinct dolomite-rich bands.

Based on calcite-dolomite ternary solvus thermometry^39^ an average temperature of 725 °C was estimated for the marble outside the SZ. Inside the SZ core the average temperature was calculated as 530 °C (cf. Supplementary Table S2).

The major-element composition is characterised by high amounts of CaO and a substantial content of SiO_2_, MgO, Fe_2_O_3_, Al_2_O_3_ and K_2_O (cf. Supplementary Table S3). Compared to pure marble, the grey band shows 2–3 times higher amounts of major-elements except for Ca. The whole-rock major-element analyses have been recalculated to modal proportion of the main mineral phases (calcite, dolomite, quartz, muscovite and kaolinite) based on the measured chemical composition of individual mineral phases. The amount of accessory phases is higher in the grey marble. Dolomite is present in all analysed samples (0.67 to 2.3%). Other present phases are quartz (0.05–2.71%), muscovite (0.03–0.65%) and kaolinite (0.03–0.28%) (cf. Supplementary Table S4).

### Microstructure

**Figure 3:**
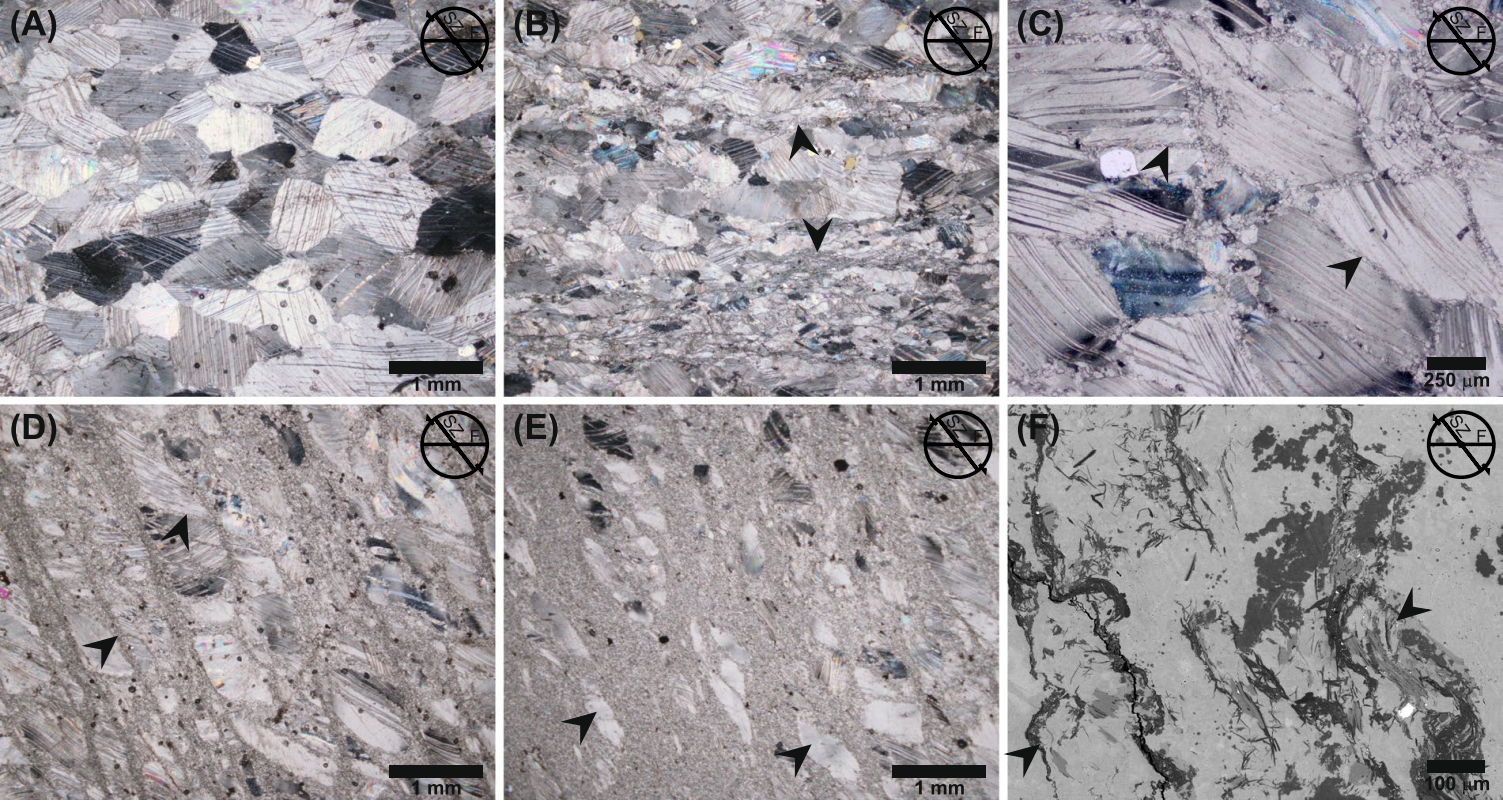
Micrographs of microstructural evolution across the shear zone. Orientation of all micrographs is marked in their top-right corner (SZ - shear zone, F- foliation). (**A**) The coarse-grained microstructure of the white pure calcite band away from the shear zone. (**B**) The microstructure of the grey layer with tiny bands of accessory phases distal to the shear zone. (**C**) Onset of dynamic recrystallization along grain boundaries and cleavage planes 6 cm from the shear zone core at γ_local_ ~0.3. (**D**) Microshears of recrystallized grains that developed preferentially along grain boundaries and cleavage planes. (**E**) Porphyroclastic microstructure and completely recrystallized fine-grained microstructure in the core of the SZ. (**F**) SEM image of the folded thin band contaminated by other phases in shear zone core.

The calcite microstructures and crystallographic preferred orientation (CPO) vary according to the distance to the SZ core. The original microstructure away (>10 cm) from the SZ is formed by equilibrated calcite characterised by closely spaced e{102} twin lamellae, straight grain boundaries, 120° triple points and weak shape preferred orientation (SPO) (Fig. 3A). Calcite grainsize in the grey band is reduced by the presence of other mineral phases, such as mica that exhibits SPO parallel to foliation (Fig. 3B). At 6 cm from the SZ core (γ_local_ = 0.3) calcite starts to dynamically recrystallize along grain boundaries and cleavage planes forming core and mantle microstructure (Fig. 3C). The main expression of increasing strain agrees with the rising proportion of dynamically recrystallized grains (Fig. 3C–E). The recrystallized grains become organized in microshears developed preferentially along grain boundaries and cleavage planes (Fig. 3D). The closer to the SZ core, the higher the number and width of microshears. At 2.5 cm from the SZ core (γ_local_ = 2) the microshears start to define two preferred orientations parallel and inclined ~120° to SZ with the former becomes dominant approaching the SZ core. The recrystallization process leads to the development of porphyroclastic microstructure and formation of completely recrystallized fine-grained bands in the SZ core (Fig. 3E). As the SZ core is approached, the size of porphyroclasts decreases, the aspect ratio increases and, SPO becomes parallel to the SZ plane. In the SZ core, the thin grey band exhibits a weak preferred orientation of other phases in the very fine-grained calcite matrix due to the folded character of the band (Fig. 3F).

**Figure 4:**
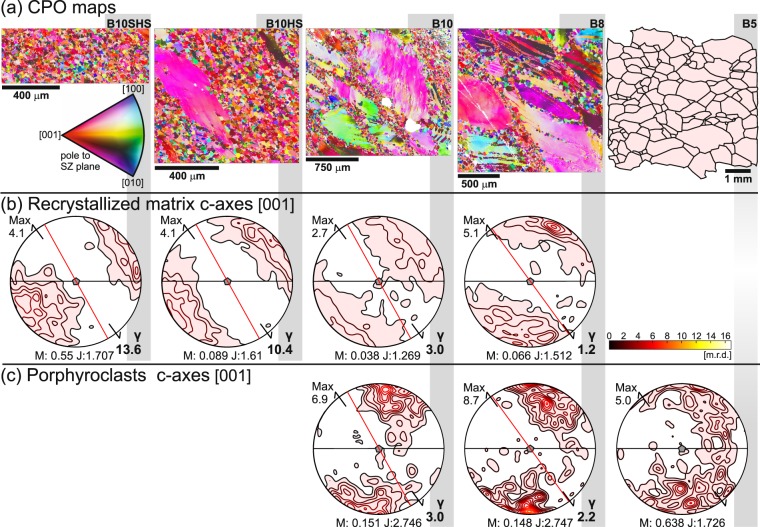
Calcite crystallographic preferred orientation (CPO) evolution across the shear zone represented by c-axes. (**a**) CPO maps of B8, B10 samples and B10 high strain (HS) and B10 super high strain (SHS) sub-parts are coloured based on the inverse pole figure of the pole to the shear zone, the coarse-grained sample B5 is represented by digitized microstructure from the micrograph. (**b**) Calcite c-axes preferred orientation of the recrystallized matrix from CPO maps. The horizontal black line is the orientation of primary foliation, the red line represents the macroscopic fabric at the sampling locality, the shear zone orientation is marked by black arrows at the pole figure rim, centre of the pole figure is the intersection of the primary foliation and the shear zone. (**c**) Calcite c-axes preferred orientation of porphyroclasts in samples B8 and B10 and primary microstructure in sample B5. Maximum (Max) of multiples of random distribution (m.r.d.) are noted for each pole figure. Contours levels are 0.7 of m.r.d. Misorientation index M-index^59^ (M), textural index^60^ (J) and local strain (γ_local_) for recrystallized grains and average strain (γ_average_) for recrystallized porphyroclast are noted for each sample.

The calcite CPO outside the SZ, exhibits incomplete girdle of c-axes with maxima normal to foliation and weak broad girdle of <a>-axes subparallel to foliation (Fig. 4, Supplementary Fig. S2). With increasing strain, the CPO of recrystallized grains as well as the CPO of porphyroclasts progressively evolves. The CPO of porphyroclasts affected by recrystallization in microshears (B8, γ_average_ = 2.2) is similar in orientation to that observed away from the SZ (Fig. 4, Supplementary Fig. S2) with only stronger c-axes concentration and better-defined girdle of <a>-axes. At higher strain (B10, γ_average_ = 3.0), a substantial population of porphyroclast c-axes shows orientation perpendicular to the SZ plane (Fig. 4, Supplementary Fig. S2). The recrystallized grains in microshears are characterised by the CPO pattern of single c-axes concentration and perpendicular <a>-axes girdle, inclined 25° to the primary coarse-grained foliation at low strain (γ_local_ = 1.2, Fig. 4, Supplementary Fig. S2). The angular deviation between the CPO pattern and foliation increases with strain to ~65° at maximal strain (γ_local_ = 13.6), where the microstucture is completely recrystallized. The calcite CPO in the recrystallized thin grey micro bands is similar to the CPO of the pure calcite. Only the CPO is slightly weaker (Fig. 4, Supplementary Fig. S2).

### Rock magnetism

**Figure 5:**
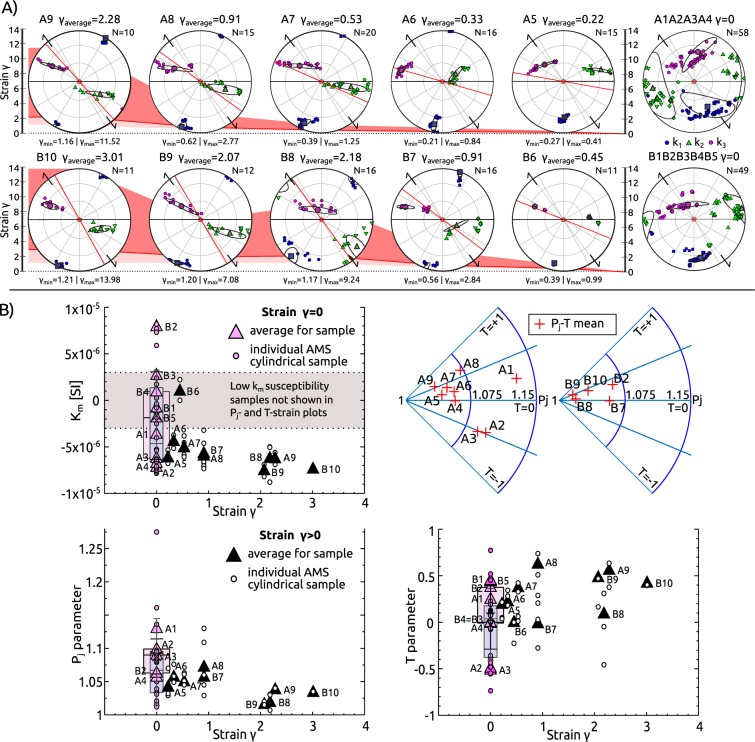
Evolution of anisotropy of magnetic susceptibility (AMS) across the shear zone. (**A**) AMS orientation from both profiles A and B with increasing strain (γ_average_) from right to left. In the image background strain is visualised by coloured fields (γ_average_ - red line, max γ_local_ - top of the red field and min γ_local_ - bottom of pink field). Diagrams are in the same structural framework as in Fig. 4. (**B**) Diagrams on magnetic parameters variation: P_j_ - T polar plots for profile A and B; k_m_ vs. strain (γ_average_); P_j_ parameter vs. γ_average_; T parameter vs. γ_average_, both mean values for the samples and individual cylindrical specimens are shown. Datapoints for γ_average_ = 0 are represented also by boxplots: pink - from the samples, blue - from individual specimens.

Mean magnetic susceptibility k_m_ values are very low for the two investigated profiles, ranging from −7.8 × 10^−6^ to 2.4 × 10^−6^ [SI] in profile A, with most of the specimens very close to the value of a single calcite crystal, and −8.8 × 10^−6^ to 8.2 × 10^−6^ [SI] in the profile B (Fig. 5B). Along the strain profiles, k_m_ in samples affected by SZ related deformation generally decreases from weakly diamagnetic and even paramagnetic samples to strongly diamagnetic samples in the SZ core. The thermomagnetic curves at the whole temperature interval have a noisy character showing weak linear decrease of magnetic susceptibility with increasing temperature (Supplementary Fig. S3).This is characteristic for dominant diamagnetic material^40^ likely accompanied by minor amounts of paramagnetic phases. Hysteresis loops show linearly correlated magnetization with the applied field, revealing only a negligible concentration of ferromagnetic components (Supplementary Fig. S3), which is supported by the character of isothermal remanent magnetization (IRM) and DC demagnetization (DCD) curves (Supplementary Fig. S3).

### AMS fabric

AMS data^41^ show in general well-defined fabric patterns that are similar in both profiles. In the undeformed region (A1-A4 and B1-B5), magnetic lineation (k_1_) is oblique to the macrostructural foliation (almost perpendicular to foliation in the B profile, Fig. 5). AMS axes k_2_ and k_3_ form a weak girdle, which is inclined to macro foliation with a more pronounced girdle and a smaller angular deviation in profile B. Further along the profile towards the centre of the SZ, the magnetic fabric is characterised by clustering of k_1_ axes close to the macro foliation pole and spreading of k_2_ and k_3_ axes into a girdle subparallel to the macro foliation. This pattern shows a gradual increase of the angular deviation with respect to the local macro fabric, from about 10° in samples A5, B6 to ~30° in samples A9, B10.

Distal to the SZ core, the degree of AMS (P_j_ parameter) is small and variable. Approaching the SZ, the P_j_ parameter decreases and the shape parameter T evolves from neutral (or even prolate shape - samples A2, A3) to the oblate shapes achieved in the SZ core.

## Discussion

Deformation related to the SZ development is characterised by the build-up of a dynamically recrystallized calcite matrix at the expense of porphyroclasts and complete recrystallization in the core of the SZ. The microstructural evolution of both recrystallized calcite matrix and porphyroclasts is reflected in distinct CPO patterns. Their respective calcite c-axes orientations display an angular deviation that increases with strain. Results obtained along the strain profile show the k_1_ AMS axes parallel to calcite c-axes preferred orientation but at a high angle to the local macro foliation. The AMS pattern is characterised by the k_1_ axes being at a high angle to foliation and a girdle distribution of k_2_ and k_3_ axes being subparallel to foliation, which gradually rotates clockwise with increasing strain. Moreover, the angle between the orientation of the local macro foliation pole and k_1_ axes also increases with strain.

The first observation that needs to be clarified is the discrepancy between the relationship of the measured and the expected AMS as defined by the calcite single crystal AMS tensor. The single crystal is diamagnetic with the k_3_ axis parallel to its crystallographic c-axis and k_1_ and k_2_ axes within the plane of the crystal <a>-axes^42^. Accordingly, AMS in the pure calcite marble should be defined by the k_3_ axis and be perpendicular to the macroscopic foliation corresponding to the preferred c-axes orientation^43^ (Fig. 4), what isn’t observed in these samples.

This relationship between the AMS and macroscopic planar fabric has been termed an inverse AMS fabric^44,45^. The origin of the inverse AMS fabric has been previously attributed to several causes: single-domain (SD) state of tiny ferromagnetic particles^46^; microstructural arrangement of AMS carriers by a particular type of flow/deformation of rock^47^; and/or the chemical composition of constituent rock forming minerals such as the content of paramagnetic elements in the mineral lattice and oxidation state of these elements^48^. However, high field measurements of hysteresis loops, IRM acquisition curves and DCD exclude the possible occurrence of SD particles (Supplementary Fig. S3) as carrier of the inverse fabric. Neither, any microstructural arrangement that could explain the inverse fabric has been identified. On the other hand, the whole rock and mineral chemical composition disclosed a considerable FeO amount both in the white and the grey contaminated marble bands. From now, “inverse” notion is adopted to differentiate between an inverse fabric due to the granulometric state of magnetic particles and an “inverse” fabric related to structural framework.

**Figure 6:**
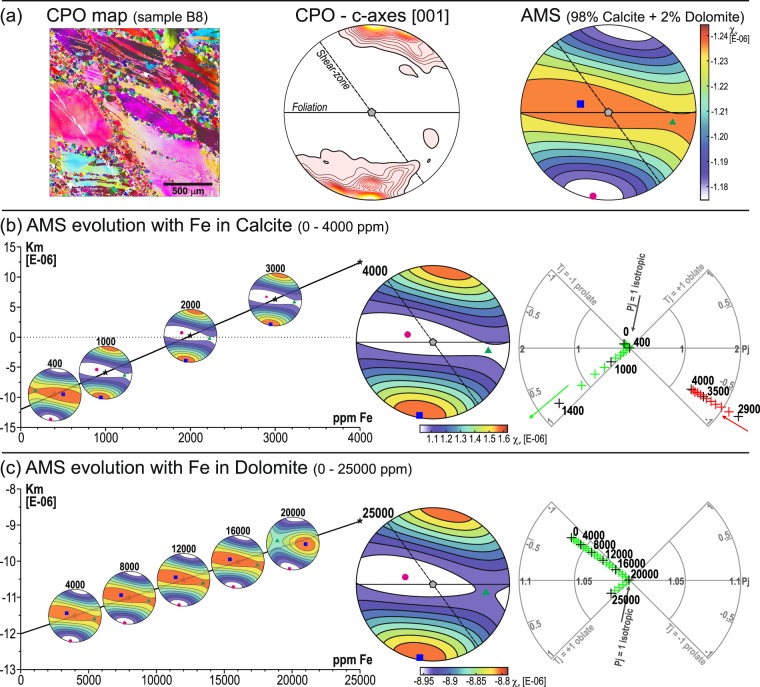
Results of numerical modelling of “inverse” fabric origin. (**a**) The background for numerical modelling, from left to right: CPO map from sample B8, calcite c-axes preferred orientation from the CPO map and modelled AMS based on calcite and dolomite single crystal properties (98% calcite and 2% dolomite). (**b**) AMS evolution with increasing Fe content in calcite from 0 to 4000 ppm. (**c**) AMS evolution with increasing Fe content in dolomite from 0 to 25000 ppm. In (**b,c**) from left to right: graph of *k*_*m*_ vs. Fe content with modelled AMS orientations at specific Fe concentration levels, modelled AMS orientation at maximum concentration and *P*_*j*_ − *T* polar plot of modelled evolution with increasing Fe content. CPO and AMS diagrams are in the same structural framework as in Figs. 4 and 5.

To explore the role of Fe concentration in constituent minerals for the “inverse” fabrics, a set of numerical models was calculated. The CPO map obtained from the sample B8 and the composition of 98% calcite and 2% dolomite with single-crystal AMS tensors of pure calcite and dolomite were taken as a base for the AMS modelling (Fig. 6a). The first model series explore the influence of increasing content of Fe in the calcite crystals lattice (Fig. 6b). For the single-crystal AMS tensor evolution with the increasing Fe concentration, the empirical relationship described for calcite^42^ was used (for detail see Supplementary Table S5). Regarding the mean magnetic susceptibility signal, it remains negative up to 2000 ppm of Fe in the calcite crystals lattice (Fig. 6b). The axes orientations and shape of the AMS ellipsoid undergo a reversal from normal to “inverse” AMS and change from prolate to oblate shape is observed to occur between 400 and 500 ppm of Fe in the calcite crystals lattice (Fig. 6b). A similar Fe concentration in calcite was however detected only in a few calcite grains within the tiny grey band as shown by calcite composition (Supplementary Table S1) and chemical composition maps (Supplementary Fig. S1). These observations suggest that the Fe content in calcite is not a determinant factor to justify the “inverse” AMS fabric.

Another possibility to explore is the presence of Fe in the dolomite crystals. The second model series explore the effect of increasing Fe content in the dolomite. A similar empirical relationship as for the calcite was used, as the crystal structure of dolomite closely resembles that of calcite and no empirical relationship exists for dolomite^40^. The AMS evolution is characterised by a linear increase of susceptibility and stable axes orientation up to 20000 ppm of Fe in the dolomite (Fig. 6c). The transition from normal to “inverse” AMS fabric and tensor changes from prolate to oblate occurs between 20000 and 25000 ppm of Fe but still at negative mean susceptibility values (Fig. 6c). Thus, the observed “inverse” AMS fabric can be explained by about 2% dolomite with 2.5% of Fe, which is similar to the observed values. However, a small volume of a high-susceptibility/high-anisotropy mineral such as mica may contribute significantly to the magnetic fabric. Therefore, muscovite with the observed chemical composition was included in the model for sample B5. As little as 0.5% of muscovite concentration leads to a prolate fabric, a switch of the k_3_ orientation normal to the foliation and a small increase in k_m_. This is inconsistent with the measured magnetic fabric of sample B5 and suggests a lower content of Fe-muscovite in these rocks. The presence of only ~0.2% can explain the observed obliquity of AMS and primary foliation orientations in the samples external to the SZ (Supplementary Fig. S4). The modelled mean magnetic susceptibility only has good agreement with the measured values from the pure white marble. The grey marble shows higher measured susceptibility values that are likely to be caused by two possible reasons. One corresponds to the possibility of a higher dolomite content, and the other to the observed local increase of Fe content in the calcite. Nevertheless, the CPO of calcite and dolomite dominates the observed AMS.

**Figure 7:**
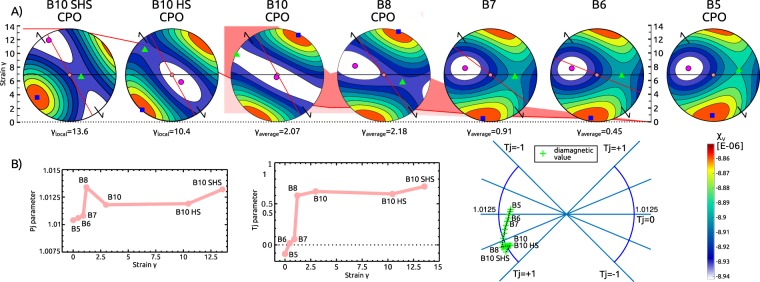
Results of numerical modelling of the AMS evolution with strain. (**A**) Calculated AMS for different samples and their evolution. Those diagrams calculated directly from CPO data are noted. Remaining diagrams are calculated based on CPO endmember combination with microstructural data. In the image background strain is visualised in the same way as in Fig. 5. AMS diagrams are in the same structural framework as in Figs. 4, 5 and 6. (**B**) Diagrams of magnetic parameters variation: *P*_*j*_ parameter vs. γ_average_; *T* parameter vs. γ_average_, *P*_*j*_ − *T* polar plot. The modelled fabrics exhibit very weak AMS, with the transition from neutral/prolate to oblate shape (*T*: -0.1 → 0.6) occurring between samples B5 and B8. All medium to highly deformed samples B8 → B10SHS show stable oblate shape (*T* ~ 0.6). The intensity of the AMS within the model is very low and has to be interpreted with caution. Nevertheless, the *P*_*j*_ parameter oscillates between *P*_*j*_ = 1.0103 and *P*_*j*_ = 1.0135. It initially increases with the observed deformation from B5 to B8 (*P*_*j*_: 1.0103 → 1.0135), then decreases from B8 to B10HS (*P*_*j*_: 1.0135 → 1.0117) and increases again for the most deformed sample B10SHS (*P*_*j*_ = 1.013).

Now that we understand the source of the “inverse” AMS fabric we will focus on its evolution with increasing strain. The continuous set of numerical models has been calculated based on CPOs of variously-strained samples: B5, B8, B10 (B5 γ~0, B8 γ_average_~2.18, B10 γ_average_~3.01) and mineral and chemical composition (2% dolomite with 2.5% of Fe) representative of the “inverse” fabric (Fig. 7), resulting in a constant mean magnetic susceptibility of *k*_*m*_ = −8.89 × 10^−6^ [SI]. The model was calculated in steps, gradually combining distinct CPO sub-fabrics of porphyroclasts and recrystallized matrix. Their respective proportions were inferred from microstructural analysis of selected samples and microstructurally distinct parts of highly strained sample B10 (HS high strain γ_local_~10.4 and SHS super high strain γ_local_~13.6).

The models show strong agreement between the numerical and natural magnetic fabrics for all samples, away from the SZ with primary fabric to highly deformed samples within the SZ core. The modelled AMS fabric reproduces well the measured AMS axes orientation. We also observe a shift of *k*_2_ and *k*_3_ within the shear plane in highly strained samples, interpreted as a result of the rotation of mineral grains within the SZ plane around their crystallographic c-axis. The AMS models relate the variation of the observed orientations with the measured AMS as a function of the strain intensity. Increased strain augment the angular deviation between the calculated and observed AMS pattern, and the macroscopic foliation, reaching ~30° in highly strained samples (B10). Modelled AMS axes only fits the macroscopic SZ if calculated from the B10 sub-parts (B10 HS, B10 SHS) CPOs. The trend of the shape magnetic parameter *T* with increasing strain in the numerical model and natural samples is very similar. The fabric strength parameter *P*_*j*_ exhibits very low values and indistinct trend with increasing strain. The model successfully replicates AMS for samples B6 and B7 based on their microstructural description (ratio of porphyroclasts to matrix) without corresponding CPO determination. The striking similarity between measured and modelled AMS implies that orientation and shape of natural AMS are controlled by the CPO and microstructural fabric of both calcite and dolomite.

**Figure 8:**
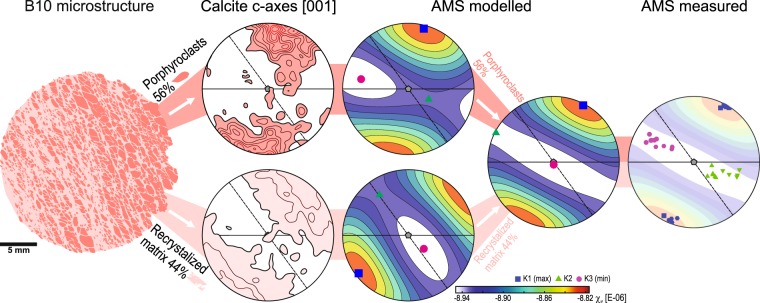
Analysis of deformation localization influence on AMS. From left to right: digitized microstructure of sample B10, calcite c-axes preferred orientation of porphyroclasts and recrystallized matrix in sample B10, modelled AMS signal of porphyroclasts and recrystallized matrix, combined modelled AMS signal from sample B10 based on the actual proportion of porphyroclasts (56%) and matrix (44%) and comparison with measured AMS in sample B10. CPO and AMS diagrams are in the same structural framework described in the. 4., 5., 6. and 7.

The microstructural record within the SZ, which is characterised by an increasing ratio of recrystallized matrix to porphyroclasts and their contrasting CPOs (Figs. 3, 4, 8), is a result of deformation localization^49^. To what extent and how this microstructure controls AMS patterns is evaluated by further numerical modelling. The model is based on the rock composition inferred for the “inverse” AMS fabric and on the recorded proportion between the recrystallized matrix (44%) and porphyroclasts (56%) and their respective CPOs in the specimen B10 of γ_average_~3.01 (Fig. 8).

The modelled AMS for matrix and porphyroclast closely mimics their respective recorded c-axes patterns, characterised by the k_1_ orientation corresponding to c-axes maximum concentration (Fig. 8). The *k*_1_ axis of porphyroclasts is 14° oblique to the pole of the primary foliation (Fig. 8), resulting from the combination of porphyroclasts that keep the primary CPO and porphyroclasts rotated towards the orientation of c-axes perpendicular to the SZ plane. In contrast, the AMS of recrystallized matrix is characterised by *k*_1_ perpendicular to the SZ plane (Fig. 8). The modelled tensors exhibit weak AMS of oblate shape characterised by the intensity *P*_*j*_ and shape *T* parameters similar for both porphyroclast (*P*_*j*_ = 1.016, *T* = 0.60) and matrix (*P*_*j*_ = 1.013, *T* = 0.62). The AMS tensors modelled for the respective microstructural elements were combined in the observed proportions. The resulting AMS is characterised by: i) *k*_1_ located between poles of the foliation and the SZ plane, which defines a magnetic foliation between the foliation and the SZ planes (Fig. 8); ii) *k*_3_ axis position appears in the intersection of the foliation and the SZ plane and; iii) AMS ellipsoid shows a weaker anisotropy and more oblate shape (*P*_*j*_ = 1.012, *T* = 0.84). The modelled AMS orientation is in close agreement with the measured AMS orientation for the B10 specimens (Fig. 8). The *k*_1_ orientation is almost identical with the measured k_1_ orientation. The measured k_3_ and k_2_ exhibit a spread between modelled *k*_2_ and *k*_3_ orientations for matrix and porphyroclast with a preference to the later. The mean modelled magnetic susceptibility *k*_*m*_ is slightly lower (−8.89 × 10^−6^ [SI]) than measured (−7.36 × 10^−6^ [SI]). The higher k_m_ and P_j_, and lower T of the measured AMS suggest a higher dolomite content or higher Fe content in the dolomite. Increasing any of them in the model would result in a less oblate AMS ellipsoid, and increases of susceptibility and anisotropy strength.

The localization of deformation at P-T conditions of dislocation creep leads to the contemporaneous evolution of two microstructural subfabrics in the marble. The combination of their respective magnetic signals results in distinct orientation of total AMS and local macroscopic fabric. This means that neither strain magnitude nor its orientation derived from macroscopic fabric orientation (Fig. 2) would correspond to estimates deduced from AMS. The combination of magnetic signal of distinct subfabrics also influences the shape and strength of magnetic anisotropy. Therefore, AMS parameters cannot be directly related to type and magnitude of strain. AMS rather reflect the proportion and orientation between subfabrics originating from localization of deformation, which only indirectly corresponds to the magnitude and character of deformation. The relationship between orientation and magnitude of strain and AMS ellipsoids can then be established only when based on microstructural analysis. In more general terms, the localized deformation plays a key role at all scales, from microstructure to large-scale deformation zones and from brittle to ductile conditions^50^ and even in magmatic flow of partly crystallized magma with frequent grain to grain interactions^5,51,52^. That is the effects of localization should be considered whenever interpreting the AMS of rocks where localized deformation can be expected.

## Conclusions

In the present study we derived the relationship between AMS and strain in a marble shear zone by combining rock magnetic studies, detailed microstructural analysis and CPO-based numerical modelling of the AMS. AMS ellipsoids are characterised by k_1_ orientation as being at a high angle to the primary fabric and experience gradual rotation towards the SZ plane with increasing strain. The k_1_ orientation is consistent with the calcite c-axes preferred orientation, which is considered to represent an “inverse” AMS fabric, because the “normal” AMS fabric should show k_3_ parallel to c-axes. Moreover, the AMS shows an angular deviation from the local macroscopic fabric observed in the shear zone. The microstructural evolution related to the shear zone development is characterised by dynamic recrystallization of a primary coarse-grained calcite microstructure. The increasing strain is accommodated by increasing the amount of recrystallized matrix at the porphyroclasts expense.

The calcite CPO based numerical modelling of the AMS revealed the source of the observed “inverse” AMS fabric in the presence of Fe-dolomite (>=2%). This suggests that separation between paramagnetic and diamagnetic signals at low temperature could by an useful tool to examine the composite AMS fabrics^53^. Another set of numerical models documented a close correlation between measured and modelled AMS orientations while examining the relationship to strain. Therefore, we conclude that the CPO of calcite and dolomite subfabrics controls the AMS in the studied marbles. Finally, the angular deviation between the AMS and macroscopic fabric in the shear zone is explained by localization of deformation during the shear zone development with dislocation creep conditions. The shear deformation in the marble results in two distinct CPO subfabrics of recrystallized matrix and porphyroclasts. AMS integrates the magnetic signature of these subfarics and therefore cannot be directly correlated with the orientation and magnitude of the strain ellipsoid. The assessment of the indirect relationship between the finite strain and the AMS ellipsoid in rocks prone to localization of deformation or flow will be significantly improved when combined with detailed microstructural analysis and knowledge of the whole rock and mineral chemical composition. Moreover, this approach has potential for inverse evaluation of unknown CPO from the character and strength of the magnetic fabric and knowledge of rock microstructural evolution. Since the localization of deformation is a multiscale process it is strongly recommended that AMS be complemented by microstructural analyses for the description of deformation or flow.

## Methods

### Sampling

The 9 samples along the profile A and 10 samples along the profile B were taken using a portable drilling machine. Each drillcore was cut into 2 to 3 standard AMS cylindrical specimens 25.4 mm in diameter and 22 mm long.

### Mineral chemical composition

The quantitative chemical analyses of calcite, dolomite and muscovite were acquired at the Laboratory of Scanning Electron Microscopy and Microanalysis of the Institute of Petrology and Structural Geology (Faculty of Science, Charles University, Prague). The quantitative analyses were completed using a Scanning Electron Microscope coupled with Energy Dispersive Spectroscope (SEM-EDS). The instrument used was a Tescan Vega SEM equipped with EDS X-Max 50 (Oxford Instruments), 15 kV accelerating voltage and 1.5 nA beam current.

### X-ray diffraction

The samples A2/1, A5/3, A9/1, A9/2, B2/2, B6/2, B8/2 were inspected using a Bruker D-8 DISCOVER X-ray powder diffractometer. This is a multi-purpose powder X-ray diffraction instrument with a variable measuring radius located at the Institute of Geology of the Czech Academy of Sciences in Prague.

### Chemical compositional maps

The compositional maps were acquired with a Field Emission Gun Electron Probe Microanalyser (FEG-EPMA, JXA-8530F by Jeol) with 25 kV accelerating voltage and 70 nA probe current at the Laboratory of Microprobe Analysis of the Institute of Petrology and Structural Geology of the Faculty of Science, Charles University, Prague.

### Whole-rock major- and trace-element chemical composition

The whole-rock major- and trace-element analyses of marbles were determined in the Bureau Veritas Commodities Canada Ltd., Vancouver, by a combination of inductively coupled plasma-optical emission spectrometry (ICP-OES) and inductively coupled plasma-mass spectrometry (ICP-MS).

### Microstructural observation (EBSD)

The marble microstructure was observed by optical microscopy of thin sections cut perpendicular to the drilled AMS cylinders’ axis. The calcite crystallographic preferred orientation (CPO) was acquired by means of the Electron Back Scattered Diffraction (EBSD) method in the Laboratory of Scanning Electron Microscopy and Microanalysis of the Institute of Petrology and Structural Geology (Faculty of Science, Charles University, Prague). The EBSD measurements were performed with the Vega Tescan SEM equipped by NordlysNano EBSD camera (Oxford Instruments) operated at 20 kV accelerating voltage and 7 nA probe current in mapping mode for samples B8 and B10 and manually to acquire the CPO of coarse grains in samples B5, B8 and B10. The acquired EBSD maps and manual data were processed using the MTEX 4.5.0 MATLAB toolbox^54^.

### Rock/mineral magnetism

Thermomagnetic and high-field experiments (classical hysteresis, Isothermal Remanent Magnetization – IRM and Direct Current Demagnetization – DCD measurements) were conducted in order to specify the type of the main magnetic carriers, their grain size state and concentration. The dependence of susceptibility with temperature was investigated using the KLY–4 S Kappabridge coupled with CS-3 Furnace and CS-L Cryostat apparatus with sensitivity 3 × 10^−8^ [SI] at the Institute of Geophysics, Academy of Sciences of the Czech Republic. The measured data were processed using the computer code Cureval (AGICO, Inc.). The susceptibility variations with temperature were measured from −196 °C to 5 °C and between room temperature and 700 °C (heating and cooling curves). Classical hysteresis and remanent parameters were measured by a Vibrating Sample Magnetometer (Model EV9 VSM, DSM Magnetics; ADE Corporation, Lowell, MA, USA) on two specimens from both marble types.

The AMS was measured with the MFK1 Kappabridge apparatus with sensitivity of 2 × 10^−8^ [SI] at the Institute of Geophysics, Academy of Sciences of the Czech Republic. Each specimen was measured five times to increase the precision of the AMS determination^55^. A statistical analysis of the AMS data was carried out using the ANISOFT package of programs (written by M. Chadima and V. Jelínek; www.agico.com). The AMS data are represented by the k_m_, P_j_, and T parameters defined as follows: (1) k_m_ = (k_1_ + k_2_ + k_3_)/3; (2) ln(P_j_) = ln(P) * (1 + (T^2^)/3)^1/2^; and (3) T = 2ln(k_2_/k_3_)/ln(k_1_/k_3_) − 1; where k_1_ ≥ k_2_ ≥ k_3_ are the principal magnetic susceptibilities and P = k_1_/k_3_. The parameter k_m_ represents the mean magnetic susceptibility. The parameter P_j_, called the degree of AMS^56^, reflects the eccentricity of the AMS ellipsoid and thus indicates the intensity of the preferred orientation of the magnetic minerals in the rock. The parameter T characterises the shape of the AMS ellipsoid^55^; it varies from −1 (perfectly linear magnetic fabric) through 0 (transition between linear and planar magnetic fabric) to +1 (perfectly planar magnetic fabric). In fabric studies, the maximum principal susceptibility (k_1_) is referred to as the magnetic lineation, and the minimum principal susceptibility (k_3_) defines a pole to the magnetic foliation; their orientations are presented in stereograms in the specimen, structural or geographical coordinate system. We followed the experienced practice of the AGICO programs dealing with diamagnetic samples: the first approach (signed negative values) for plotting principal directions and second approach (absolute values) in calculating all AMS parameters^57^.

### AMS - Numerical modelling

The second rank tensor of magnetic susceptibility (i.e. anisotropic physical property) was calculated from texture data using the MTEX open source package^58^ using the weighted summation for individual orientation EBSD data and single crystal magnetic tensor properties. Single or polycrystalline materials of single phase or multiphase aggregates can be investigated by the Voigt, Reuss and Hill methods for calculating average physical properties. For the purposes of this paper we considered results from the Voigt method, in as much as the results from all three methods are similar.

The AMS tensor for a pure calcite single crystal is defined with values k_1_ = k_2_ = −11.723 × 10^−6^, k_3_ = −12.823 × 10^−6^ [SI] and mean k_m_ = −12.08 × 10^−6^ [SI]^42^. Pure calcite is diamagnetic with k_3_ susceptibility axis parallel to its crystallographic c axis and k_1_ and k_2_ axes in the plane of the crystal a axes. The AMS tensor for a pure dolomite single crystal is defined with values k_1_ = −7.654 × 10^−6^, k_2_ = −8,037 × 10^−6^ and k_3_ = −8.676 × 10^−6^ [SI] and mean k_m_ = −8,1224 × 10^−6^ [SI]^40^ (samples D2, D3, LF only). Pure dolomite is diamagnetic with k_1_ susceptibility axis parallel to its crystallographic a axis and k_3_ susceptibility axis parallel to its crystallographic c axis. Single crystal magnetic tensor properties of carbonate crystal are dependent on chemical composition. With respect to magnetic susceptibility and its anisotropy, the most important is substitution of calcium cation by iron (and manganese) ions. The empirical relationships for the paramagnetic susceptibility difference and for the increase of susceptibility with raising Fe concentration is well described for calcite^42^:

$${\Delta k}_{{\rm{para}}}[{{\rm{m}}}^{3}/{\rm{kg}}]={\rm{Fe}}\,{\rm{content}}\,[{\rm{ppm}}]\times (1+-\,0.1)\times {10}^{-12}[{{\rm{m}}}^{3}/{\rm{kg}}/{\rm{ppm}}];$$

increase of susceptibility is 2.3 × 10^−10^ [m^3^/kg] per 100 ppm Fe.

A similar relationship is missing for dolomite, but expecting the same substitution of Fe for Ca in dolomite, we adopted this description for dolomite also. This allows the numerical modelling of the AMS tensor for any combination of pure calcite, pure dolomite, Fe-rich calcite and Fe-rich dolomite. In general, paramagnetic iron-rich calcite/dolomite has an “inverse” magnetic fabric with the k_1_ axis coinciding with the c-axis of the crystal lattice. From the calculated magnetic susceptibility tensor, all magnetic fabric parameters are calculated.

## Supplementary information

Supplementary information for Localization effect on AMS fabric revealed by microstructural evidence across small-scale shear zone in marble

## Data Availability

AMS data and EBSD maps datasets generated and analysed during the current study are available in the World Data Center PANGAEA repository^41^.
