# Supplementary material for: Localization effect on AMS fabric revealed by microstructural evidence across small-scale shear zone in marble
Source: Sci Rep. 2019 Nov 25;9:17483. doi: 10.1038/s41598-019-53794-y (PMC6877632; doi:10.1038/s41598-019-53794-y)
Supplement: Supplementary file 1 — Supplementary information for Localization effect on AMS fabric revealed by microstructural evidence across small-scale shear zone in marble [file 41598_2019_53794_MOESM1_ESM.pdf]

Supplementary information for

Localization effect on AMS fabric revealed by microstructural evidence across small-scale shear zone in marble

Kusbach, V. *, Machek, M., Roxerová, Z., Racek M., Silva, P.F.
*To whom correspondence should be addressed: kusbach@ig.cas.cz

This pdf file includes:

Supplementary Table S1 | Mineral chemical composition from SEM-EDS. Sample B5 outside of shear zone, sample B10 is from the shear zone core.

Supplementary Table S2 | Calcite-dolomite ternary solvus thermometry based on the mole fraction of $MgCO_3$ and $FeCO_3$ in calcite.

Supplementary Table S3 | Major- and trace-element whole-rock geochemical analyses from the Estremoz marble shear zone (wt. % and ppm).

Supplementary Table S4 | Modal composition of studied samples calculated from WR chemical compositon and average mineral composition.

Supplementary Table S5 | Magnetic susceptibility tensors.

Supplementary Figure S1 | Relative chemical compositional maps of microstructurally different samples.

Supplementary Figure S2 | Calcite CPO details evolution across the shear zone.

Supplementary Figure S3 | Magnetic properties of studied samples.

Supplementary Figure S4 | AMS data with confidence ellipses for each measurement.

Supplementary Figure S5 | Results of numerical modelling of muscovite contribution to magnetic fabric.

Supplementary Table S1 | EDS mineral chemical composition data (wt. %)

Mineral Spot	Na_2O (wt.%)	MgO (wt.%)	Al_2O_3 (wt.%)	SiO_2 (wt.%)	K_2O (wt.%)	CaO (wt.%)	TiO_2 (wt.%)	FeO (wt.%)	F (wt.%)	Total (wt.%)
calcite										
B5-1-13	0.00	1.29	0.00	0.00	0.00	50.70	0.00	0.29	0.00	52.28
B5-1-24	0.00	1.17	0.00	0.00	0.00	51.09	0.00	0.00	0.00	52.27
B5-1-9	0.00	1.40	0.00	0.00	0.00	51.10	0.00	0.35	0.00	52.85
B5-1-27	0.00	1.22	0.00	0.00	0.00	51.55	0.00	0.15	0.00	52.93
b5-1-29	0.00	1.33	0.00	0.00	0.00	51.64	0.00	0.00	0.00	52.96
B5-1-1	0.00	1.21	0.00	0.00	0.00	51.92	0.00	0.17	0.00	53.29
B5-1-26	0.00	0.89	0.00	0.00	0.00	51.98	0.00	0.00	0.00	52.87
b5-1-25	0.00	0.89	0.00	0.00	0.00	52.11	0.00	0.16	0.00	53.16
B5-1-28	0.00	0.76	0.00	0.00	0.00	52.24	0.00	0.00	0.00	53.00
b5-1-30	0.00	0.78	0.00	0.00	0.00	52.29	0.00	0.00	0.00	53.07
B5-1-8	0.00	1.38	0.00	0.00	0.00	52.37	0.00	0.33	0.00	54.08
B5-1-21	0.00	0.48	0.00	0.00	0.00	52.61	0.00	0.00	0.00	53.09
B5-1-2	0.00	1.14	0.00	0.00	0.00	52.81	0.00	0.13	0.00	54.08
B5-1-22	0.00	0.58	0.00	0.00	0.00	53.17	0.00	0.00	0.00	53.74
B5-1-16	0.00	1.03	0.00	0.00	0.00	53.31	0.00	0.27	0.00	54.60
B5-1-31	0.00	1.07	0.00	0.00	0.00	53.38	0.00	0.00	0.00	54.46
B10-1-2	0.00	1.28	0.00	0.37	0.00	51.21	0.00	0.16	0.00	53.02
B10-1-20	0.00	0.37	0.00	0.45	0.00	52.01	0.00	0.00	0.00	52.83
B10-1-5	0.00	0.47	0.00	0.46	0.24	52.48	0.00	0.15	0.00	53.81
B10-1-21	0.00	1.03	0.00	0.54	0.00	52.49	0.00	0.00	0.00	54.06
B10-1-17	0.00	0.51	0.00	0.31	0.00	52.51	0.00	0.12	0.00	53.45
B10-1-10	0.00	0.60	0.00	0.38	0.00	52.77	0.00	0.00	0.00	53.75
B10-1-19	0.00	0.61	0.00	0.46	0.00	53.09	0.00	0.00	0.00	54.16
B10-1-12	0.00	0.52	0.00	0.45	0.00	53.17	0.00	0.21	0.00	54.36
B10-1-18	0.00	0.53	0.00	0.62	0.00	53.29	0.00	0.00	0.00	54.44
B10-1-11	0.00	0.46	0.00	0.36	0.00	53.66	0.00	0.00	0.00	54.47
B10-1-16	0.00	0.31	0.00	0.40	0.00	54.45	0.00	0.00	0.00	55.16
dolomite										
B5-1-7	0.00	18.28	0.00	0.00	0.00	29.03	0.00	2.09	0.00	49.40
B5-1-12	0.00	18.07	0.00	0.00	0.00	29.36	0.00	1.78	0.00	49.21
B5-1-15	0.00	18.46	0.00	0.39	0.00	29.46	0.00	2.23	0.00	50.54
B5-1-5	0.00	18.38	0.00	0.00	0.00	29.84	0.00	2.07	0.00	50.29
B10-1-15	0.00	18.13	0.00	0.45	0.00	28.72	0.00	1.77	0.00	49.07
B10-1-22	0.00	19.02	0.00	0.38	0.00	28.85	0.00	1.57	0.00	49.82
B10-1-9	0.00	18.73	0.00	0.34	0.00	28.99	0.00	1.41	0.00	49.48
B10-1-3	0.00	19.08	0.00	0.38	0.00	28.99	0.00	1.49	0.00	49.95
B10-1-7	0.00	18.96	0.00	0.39	0.00	29.22	0.00	1.46	0.00	50.03
mica										
B5-1-3	0.15	3.55	27.23	49.44	11.42	0.14	0.67	2.78	0.00	95.38
B5-1-6	0.17	3.29	26.39	47.95	11.23	0.20	0.73	2.61	0.00	92.56
B5-1-4	0.14	3.05	27.83	48.34	11.47	0.21	0.94	2.76	0.00	94.75
B5-1-10	0.15	2.50	29.74	47.16	11.36	0.23	1.22	2.46	0.00	94.82
B5-1-17	0.21	2.33	29.37	46.28	11.34	0.25	0.97	3.23	0.00	93.98
B5-1-20	0.23	2.08	28.76	45.12	10.96	0.25	1.10	3.93	0.00	92.43
B5-1-11	0.19	3.23	28.16	46.38	11.24	0.30	0.98	2.45	0.00	92.93
B5-1-23	0.19	1.91	30.19	45.56	10.92	0.34	1.12	4.09	0.00	94.32
B5-1-14	0.24	2.28	29.31	45.82	11.20	0.52	1.12	3.89	0.00	94.39
B10-1-1	0.06	5.30	26.13	50.81	10.96	0.14	0.38	1.43	0.24	95.45
B10-1-23	0.08	3.99	26.77	50.46	11.06	0.21	0.56	1.11	0.43	94.67
B10-1-13	0.18	3.00	29.61	44.91	11.15	0.22	1.08	2.14	0.00	92.28
B10-1-4	0.09	3.00	27.22	46.82	11.28	0.23	0.82	1.38	0.24	91.10
B10-1-14	0.23	2.38	28.74	44.68	11.08	0.29	1.07	3.63	0.00	92.11
B10-1-8	0.19	2.30	29.26	46.15	11.25	0.37	1.01	3.47	0.00	93.99
B10-1-6	0.10	4.22	27.06	50.85	10.87	0.50	0.49	1.33	0.41	95.83
kaolinite										
B5-1-19	0.09	0.29	36.66	46.76	1.61	0.32	0.15	0.59	0.00	86.48
B10-1-18	0.00	0.24	37.62	47.05	0.00	0.47	0.00	0.00	0.00	85.38
average mineral composition in sample B5										
B5-calcite	0.00	1.04	0.00	0.00	0.00	52.14	0.00	0.12	0.00	53.30
stdev	0.00	0.28	0.00	0.00	0.00	0.80	0.00	0.13	0.00	0.70
B5-dolomite	0.00	18.30	0.00	0.10	0.00	29.42	0.00	2.04	0.00	49.86
stdev	0.00	0.17	0.00	0.19	0.00	0.33	0.00	0.19	0.00	0.65
B5-mica	0.18	2.69	28.56	46.89	11.24	0.27	0.98	3.13	0.00	93.95
stdev	0.04	0.60	1.25	1.44	0.19	0.11	0.19	0.67	0.00	1.06
B5-kaolinite	0.09	0.29	36.66	46.76	1.61	0.32	0.15	0.59	0.00	86.48
average mineral composition in sample B10										
B10-calcite	0.00	0.61	0.00	0.44	0.02	52.83	0.00	0.06	0.00	53.96
stdev	0.00	0.29	0.00	0.09	0.07	0.86	0.00	0.08	0.00	0.68
B10-dolomite	0.00	18.79	0.00	0.39	0.00	28.96	0.00	1.54	0.00	49.67
stdev	0.00	0.39	0.00	0.04	0.00	0.19	0.00	0.14	0.00	0.40
B10-mica	0.13	3.46	27.83	47.81	11.09	0.28	0.77	2.07	0.19	93.63
stdev	0.07	1.09	1.35	2.81	0.15	0.12	0.30	1.06	0.19	1.82
B10-kaolinite	0.00	0.24	37.62	47.05	0.00	0.47	0.00	0.00	0.00	85.38
average mineral composition										
calcite	0.00	0.86	0.00	0.18	0.01	52.42	0.00	0.09	0.00	53.56
stdev	0.00	0.35	0.00	0.22	0.05	0.88	0.00	0.12	0.00	0.76
dolomite	0.00	18.57	0.00	0.26	0.00	29.16	0.00	1.76	0.00	49.75
stdev	0.00	0.39	0.00	0.20	0.00	0.35	0.00	0.31	0.00	0.50
mica	0.16	3.03	28.24	47.30	11.17	0.27	0.89	2.67	0.08	93.81
stdev	0.06	0.91	1.31	2.11	0.18	0.11	0.25	0.99	0.15	1.40
kaolinite	0.04	0.27	37.14	46.91	0.81	0.39	0.08	0.29	0.00	85.93
stdev	0.06	0.03	0.68	0.20	1.14	0.10	0.11	0.42	0.00	0.77

Supplementary Table S1 | Mineral chemical composition from SEM-EDS. Sample B5 outside of shear zone, sample B10 is from the shear zone core. Average compositions for minerals in each sample are shown. Also average mineral compositions from all samples are shown. For measuring details see the methods section of the manuscript. Average chemical composition of marble constituting minerals is calculated from multiple grain analysis from two samples. Low variability shows that composition of minerals is representative for the bulk rock. All minerals contain significant amounts of anions (calcite, dolomite: CO_3^{2-}, mica and kaolinite OH^-), which have not been measured in our SEM-EDS analysis. Therefore we expect a total of ca. 56 wt.% of cations for calcite 52 wt.% for dolomite, 95 wt.% for mica and 86 wt.% for kaolinite. Our analyses meet well the expected total.

Sample	Mole fraction $CaCO_3$	$MgCO_3$	$FeCO_3$	T [°C] (Ca,Mg)	T [°C] (Ca,Mg,Fe)
γ=0					
B5-1-13	0.9617	0.0340	0.0043	726	732
B5-1-9	0.9584	0.0364	0.0051	739	746
B5-1-8	0.9600	0.0353	0.0047	733	740
B5-1-16	0.9702	0.0260	0.0038	675	682
			average T [°C]		725
γ=3					
B10-1-2	0.9642	0.0334	0.0024	723	726(*)
B10-1-5	0.9854	0.0124	0.0022	508	515
B10-1-21	0.9735	0.0265	0.0000	678	678
B10-1-17	0.9849	0.0134	0.0018	529	535
B10-1-10	0.9844	0.0156	0.0000	568	568
B10-1-12	0.9834	0.0135	0.0031	532	541
B10-1-18	0.9862	0.0138	0.0000	537	537
B10-1-11	0.9883	0.0117	0.0000	492	492
B10-1-16	0.9922	0.0078	0.0000	327	327
			average T [°C]		524

(*) probably remnant of primary fabric, not included in average value

Supplementary Table S2 | Calcite-dolomite ternary solvus thermometry based on the mole fraction of $MgCO_3$ and $FeCO_3$ in calcite[1]

[1] Anovitz, L. M. & Essene, E. J. Phase Equilibria in the System CaCO3-MgCO3-FeCO3*. *J. Petrol.* **28**, 389–415, DOI: 10.1093/petrology/28.2.389 (1987).

Analyte	Unit	**A2/1** profile A $\gamma_{average}$ = 0	**A5/3** profile A $\gamma_{average}$ = 0.22	**A9/1** profile A $\gamma_{average}$ = 2.28	**A9/2** profile A $\gamma_{average}$ = 2.28	**B2/2** profile B $\gamma_{average}$ = 0	**B6/2** profile B $\gamma_{average}$ = 0.45	**B8/2** profile B $\gamma_{average}$ = 2.18	**B8/2** REP	**B8/2** REP	**B8/2** REP	**B8/2** REP
SiO_2	%	0.42	1.21	0.61	0.85	3.43	2	0.49	0.49	0.49	0.49	0.78
Al_2O_3	%	0.14	0.34	0.21	0.21	1	0.47	0.1	0.1	0.1	0.1	0.09
Fe_2O_3	%	0.07	0.11	0.09	0.09	0.35	0.24	0.08	0.08	0.08	0.08	0.08
MgO	%	0.46	0.78	0.52	0.51	1.02	1.35	0.39	0.39	0.39	0.39	0.38
CaO	%	56.49	54.81	55.7	55.3	52.33	53.41	56.12	56.12	56.12	56.12	55.84
Na_2O	%	0.04	0.06	0.01	0.02	0.03	0.02	0.02	0.02	0.02	0.02	0.02
K_2O	%	0.02	0.09	0.04	0.02	0.38	0.18	0.02	0.02	0.02	0.02	0.02
TiO_2	%	<0.01	0.02	0.01	0.01	0.05	0.02	<0.01	<0.01	<0.01	<0.01	<0.01
P_2O_5	%	<0.01	0.01	<0.01	<0.01	0.02	0.02	<0.01	<0.01	<0.01	<0.01	<0.01
MnO	%	<0.01	<0.01	0.01	0.01	0.01	0.01	<0.01	<0.01	<0.01	<0.01	<0.01
Cr_2O_3	%	<0.002	<0.002	<0.002	<0.002	<0.002	<0.002	<0.002	<0.002	<0.002	<0.002	<0.002
Ba	ppm	6	18	9	6	41	19	7	7	7	7	8
Ni	ppm	<20	<20	<20	<20	<20	<20	<20	<20	<20	<20	<20
Sc	ppm	<1	<1	<1	<1	<1	<1	<1	<1	<1	<1	<1
LOI	%	42.3	42.5	42.8	42.9	41.3	42.2	42.7	42.7	42.7	42.7	42.7
Σ	%	99.96	99.96	99.96	99.95	99.95	99.93	99.96	99.96	99.96	99.96	99.96
Be	ppm	1	<1	<1	<1	<1	<1	<1	<1	<1	<1	<1
Co	ppm	<0.2	<0.2	<0.2	<0.2	<0.2	<0.2	<0.2	<0.2	<0.2	<0.2	<0.2
Cs	ppm	<0.1	<0.1	<0.1	<0.1	0.4	0.2	<0.1	<0.1	<0.1	<0.1	<0.1
Ga	ppm	<0.5	<0.5	<0.5	<0.5	<0.5	<0.5	<0.5	<0.5	<0.5	<0.5	<0.5
Hf	ppm	<0.1	0.1	<0.1	0.1	0.3	0.1	<0.1	<0.1	<0.1	<0.1	<0.1
Nb	ppm	<0.1	<0.1	<0.1	<0.1	0.3	0.1	<0.1	<0.1	<0.1	<0.1	<0.1
Rb	ppm	0.5	1.4	0.6	0.4	7.2	3.7	0.1	0.1	0.1	0.1	0.1
Sn	ppm	3	3	4	6	3	6	3	3	3	3	3
Sr	ppm	212.8	205.7	220.1	214.7	198	245.5	205.2	205.2	205.2	205.2	196.6
Ta	ppm	<0.1	<0.1	<0.1	<0.1	<0.1	<0.1	<0.1	<0.1	<0.1	<0.1	<0.1
Th	ppm	<0.2	<0.2	<0.2	<0.2	0.5	<0.2	<0.2	<0.2	<0.2	<0.2	<0.2
U	ppm	<0.1	<0.1	<0.1	<0.1	<0.1	<0.1	<0.1	<0.1	<0.1	<0.1	<0.1
V	ppm	<8	<8	<8	<8	<8	<8	<8	<8	<8	<8	<8
W	ppm	<0.5	<0.5	<0.5	<0.5	<0.5	0.5	<0.5	<0.5	<0.5	<0.5	<0.5
Zr	ppm	2	7.4	3.3	4.5	12.1	7.3	2	2	2	2	2.8
Y	ppm	0.5	0.8	0.7	0.6	1.2	1	0.4	0.4	0.4	0.4	0.6
La	ppm	0.6	0.7	0.7	0.8	1.5	1.3	0.8	0.8	0.8	0.8	0.7
Ce	ppm	0.9	1.6	1	1.9	3.5	3	1.1	1.1	1.1	1.1	1.1
Pr	ppm	0.14	0.22	0.16	0.2	0.39	0.33	0.12	0.12	0.12	0.12	0.14
Nd	ppm	0.4	0.8	0.6	0.7	1.5	1.3	0.5	0.5	0.5	0.5	0.5
Sm	ppm	0.07	0.12	0.11	0.08	0.24	0.25	0.05	0.05	0.05	0.05	0.06
Eu	ppm	0.04	0.04	0.02	0.03	0.06	0.04	<0.02	<0.02	<0.02	<0.02	<0.02
Gd	ppm	0.11	0.14	0.12	0.12	0.3	0.21	0.09	0.09	0.09	0.09	0.07
Tb	ppm	0.02	0.02	0.02	0.02	0.04	0.03	0.02	0.02	0.02	0.02	0.01
Dy	ppm	0.07	0.12	0.08	0.1	0.25	0.17	0.06	0.06	0.06	0.06	0.08
Ho	ppm	0.02	0.03	0.02	0.02	0.05	0.04	0.02	0.02	0.02	0.02	<0.02
Er	ppm	0.06	0.09	0.08	0.07	0.15	0.11	0.04	0.04	0.04	0.04	0.05
Tm	ppm	<0.01	0.01	<0.01	<0.01	0.02	0.02	<0.01	<0.01	<0.01	<0.01	<0.01
Yb	ppm	<0.05	0.09	0.05	0.08	0.14	0.13	<0.05	<0.05	<0.05	<0.05	<0.05
Lu	ppm	<0.01	0.01	<0.01	<0.01	0.02	0.01	<0.01	<0.01	<0.01	<0.01	<0.01
TOT/C	%	12.56	12.4	12.25	12.42	12.08	12.37	12.6	12.6	12.6	12.6	-
TOT/S	%	<0.02	<0.02	<0.02	<0.02	<0.02	<0.02	<0.02	<0.02	<0.02	<0.02	-
Mo	ppm	<0.1	<0.1	<0.1	<0.1	<0.1	<0.1	<0.1	<0.1	<0.1	<0.1	-
Cu	ppm	47.4	48.8	45.2	60.7	32.2	57.7	31.8	31.8	31.8	31.8	-
Pb	ppm	2	2.1	2.3	2.1	2.1	2.9	1.9	1.9	1.9	1.9	-
Zn	ppm	2	3	2	2	3	6	2	2	2	2	-
Ni	ppm	<0.1	2	1.4	0.7	<0.1	1.3	1.9	1.9	1.9	1.9	-
As	ppm	<0.5	<0.5	<0.5	<0.5	0.7	0.5	<0.5	<0.5	<0.5	<0.5	-
Cd	ppm	<0.1	<0.1	<0.1	<0.1	<0.1	<0.1	<0.1	<0.1	<0.1	<0.1	-
Sb	ppm	<0.1	<0.1	<0.1	<0.1	<0.1	<0.1	<0.1	<0.1	<0.1	<0.1	-
Bi	ppm	<0.1	<0.1	<0.1	<0.1	<0.1	<0.1	<0.1	<0.1	<0.1	<0.1	-
Ag	ppm	<0.1	<0.1	<0.1	<0.1	<0.1	<0.1	<0.1	<0.1	<0.1	<0.1	-
Au	ppb	<0.5	<0.5	<0.5	<0.5	<0.5	<0.5	<0.5	<0.5	<0.5	<0.5	-
Hg	ppm	<0.01	<0.01	<0.01	<0.01	<0.01	<0.01	<0.01	<0.01	<0.01	<0.01	-
Tl	ppm	<0.1	<0.1	<0.1	<0.1	<0.1	<0.1	<0.1	<0.1	<0.1	<0.1	-
Se	ppm	<0.5	<0.5	<0.5	<0.5	<0.5	<0.5	<0.5	<0.5	<0.5	<0.5	-

Supplementary Table S3 | Major- and trace-element whole-rock geochemical analyses from the Estremoz marble shear zone (wt. % and ppm). Samples A2 and A5 are outside of shear zone

and samples A9 are from the shear zone core, all A samples are within the band of pure white marble. Sample B2 is outside of shear zone and samples B6 is from the weakly deformed part of shear zone, all B samples are within the grey band of marble. For measuring details see the methods section of the manuscript.

Mineral	A2/1 %	A5/3 %	A9/1 %	A9/2 %	B2/2 %	B6/2 %	B8/2 %
calcite	96.78	93.40	96.01	95.62	86.89	89.07	97.03
dolomite	0.79	1.31	0.89	0.88	1.57	2.26	0.67
kaolinite	0.16	0.20	0.19	0.28	0.07	0.03	0.09
mica	0.03	0.16	0.07	0.04	0.65	0.31	0.03
quartz	0.05	0.86	0.19	0.65	2.71	1.78	0.26

Supplementary Table S4 | Modal composition of studied samples calculated from WR chemical compositon and average mineral composition.

Calcite magnetic susceptibility tensor[2]
(0.9697, 0, 0)<br>(0, 0.9697, 0)<br>(0, 0, 1.0607)
Calcite bulk magnetic susceptibility = -4.46×10^{-9} [m^3/kg]<br>Calcite density = 2710 kg/m^3
Dolomite magnetic susceptibility tensor[3]
(0.9424, 0, 0)<br>(0, 0.9895, 0)<br>(0, 0, 1.0681)
Dolomite bulk magnetic susceptibility = -2.84×10^{-9} [m^3/kg]<br>Dolomite density = 2860 kg/m^3
Change of susceptibility in carbonate[2]
Paramagnetic contribution to Δk from the Fe content
Δk^{para}(m^3/kg)=Fe-content(ppm)x(1±0.1)$\times10^{-12}$(m^3/kg/ppm)
Increase of susceptibility
2.3×10^{-10} [m^3/kg] per 100 ppm Fe

Supplementary Table S5 | Magnetic susceptibility tensors.

[2] Schmidt, V., Günther, D. & Hirt, A. M. Magnetic anisotropy of calcite at room-temperature. *Tectonophysics.* **418**, 63–73, DOI: 10.1016/j.tecto.2005.12.019 (2006).

[3] Schmidt, V., Hirt, A. M., Hametner, K. & Günther, D. Magnetic anisotropy of carbonate minerals at room temperature and 77 K. *Amer. Miner.* **92**, 1673–1684, DOI: 10.2138/am.2007.2569 (2007).

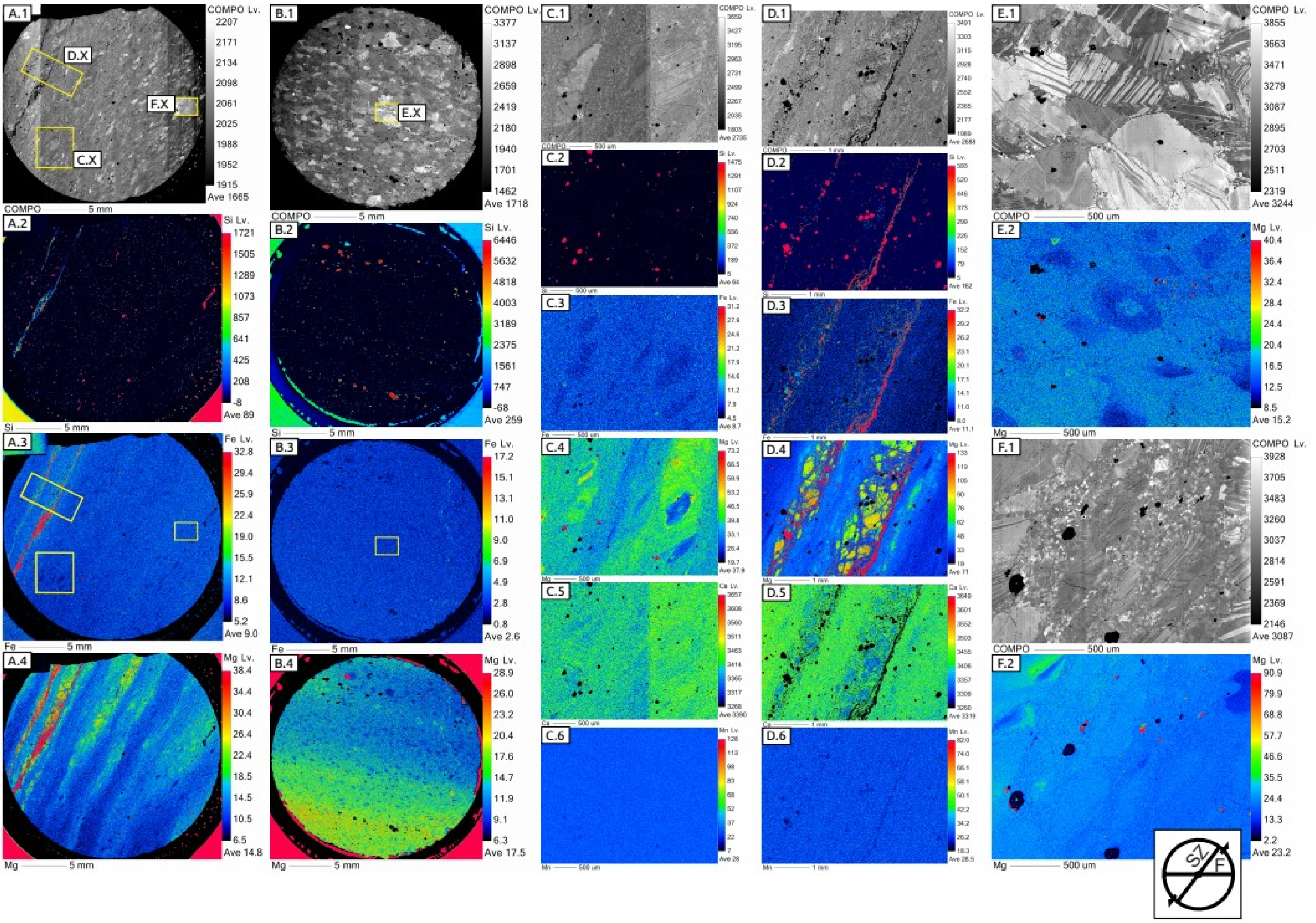

Supplementary Figure S1 | Relative chemical compositional maps of microstructurally different samples. Note the mirror orientation of the reference frame to the usually used (bottom right corner). A.X) Fine-grained B10 sample. A.1) BSE image with location of sub-maps. A.2) Si distribution within sample. A.3) Fe distribution. A.4) Mg distribution. **B.X)** Coarse-grained sample B5. B.1) BSE image with location of sub-map. B.2) Si distribution within sample. B.3) Fe distribution. B.4) Mg distribution. **C.X)** Intensivelly deformed part of sample B10, pure white marble part. C.1) BSE image. C.2) Si distribution. C.3) Fe distribution. C.4) Mg distribution. C.5) Ca distribution. C.6) Mn distribution. **D.X)** Intensivelly deformed part of sample B10, grey part, contaminated by minor phases. D.1) BSE image. D.2) Si distribution. D.3) Fe distribution. D.4) Mg distribution. D.5) Ca distribution. D.6) Mn distribution. **E.X)** Part of coarse-grained sample. E.1) BSE image. E.2) Mg distribution. **F.X)** **F**ine-grained B10 sample, part with porphyroclasts. F.1) BSE image. F.2) Mg distribution.

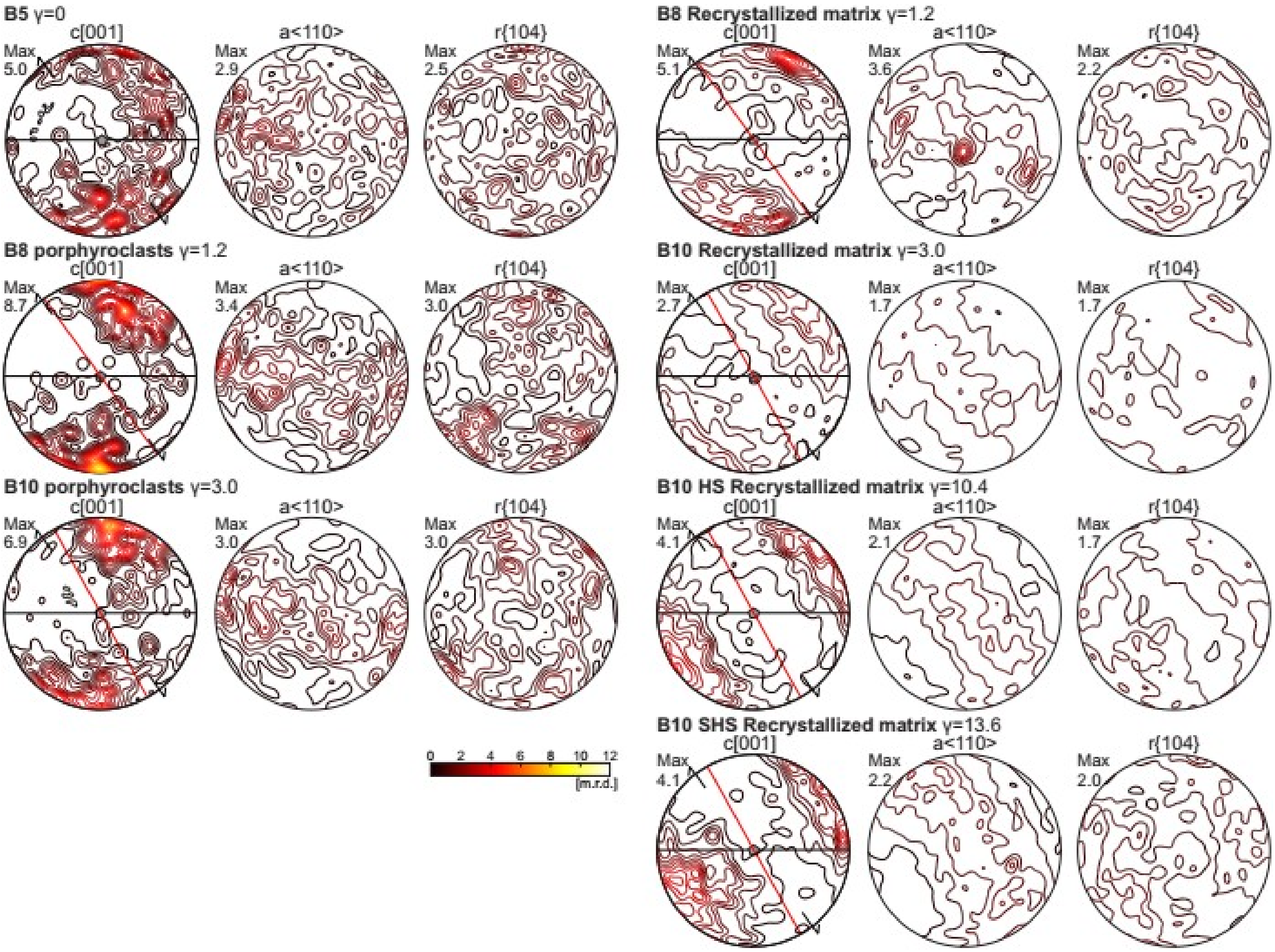

Supplementary Figure S2 | Calcite CPO details evolution across the shear zone. Represented by c<001>, a<110> axes and r{104} plane in primary fabric of sample B5 and variously-strained samples B8, B10 and subparts of B10 sample. Shown is calcite preferred orientation of porphyroclasts and of recrystallized matrix from CPO maps. The horizontal black line is the orientation of primary foliation, red line represents the macroscopic fabric at the sampling spot, the shear zone orientation is marked by black arrows at the pole figure rim, center of pole figure is intersection of primary foliation and shear zone. Maximum (Max) of multiples of random distribution (m.r.d.) are noted for each pole figure. Contours levels are 0.4 of m.r.d. Local strain (γ_{local}) for recrystallized grains and average strain ($\gamma_{average}$) for recrystallized porphyroclast are noted for each sample.

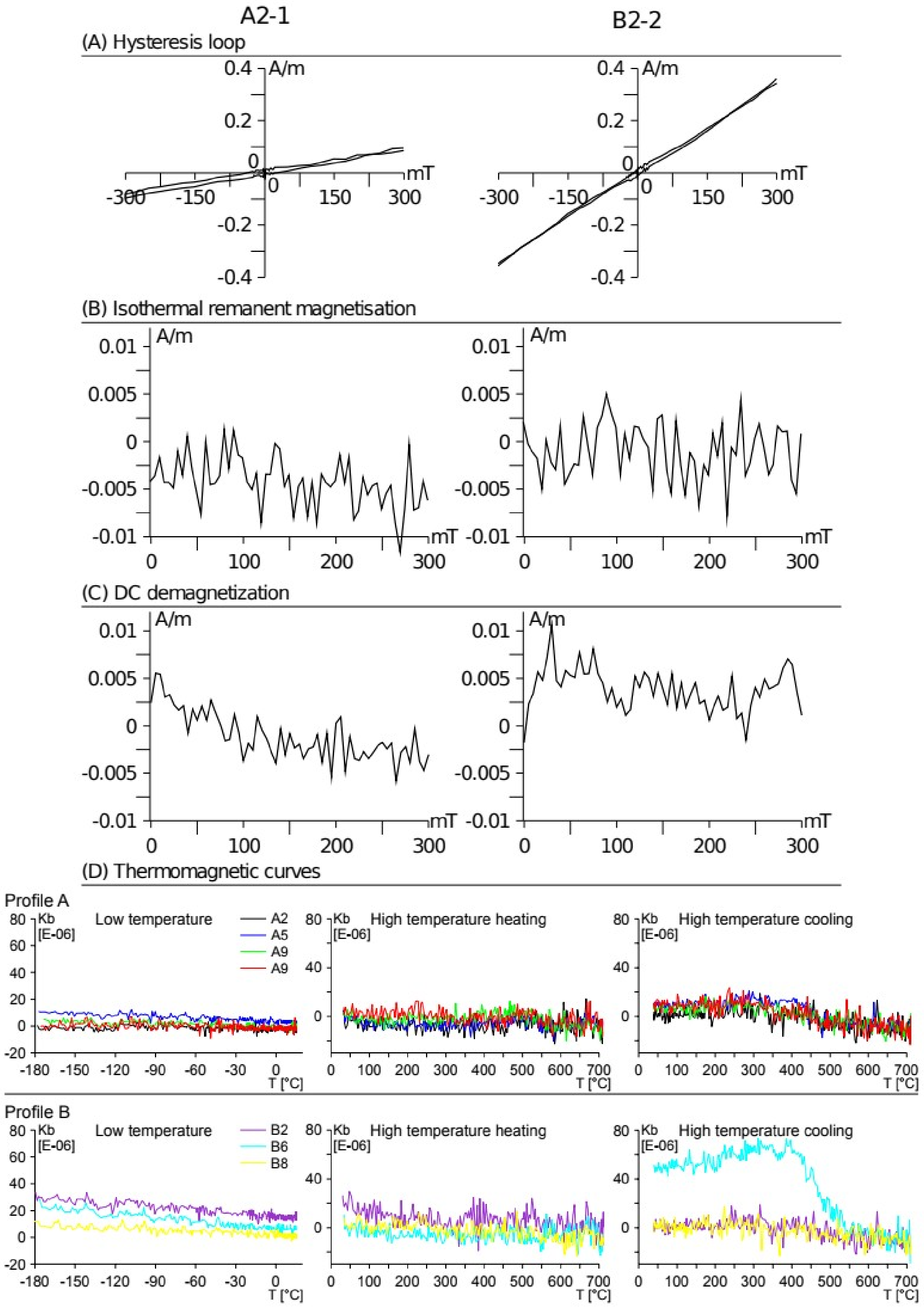

Supplementary Figure S3 | Magnetic properties of studied samples. A) Hysteresis loops. **B)** Isothermal remanent magnetization. **C)** DC demagnetization. Hysteresis loops and IRM and DCD curves show linearly correlated magnetization with the applied field, revealing only negligible concentration of ferromagnetic components. **D)** Thermomagnetic curves, the low temperature curves are not showing any significant relation between magnetic susceptibility and temperature. The measurements conducted above room temperature brings stable values with a gradual decrease between 300 and 600°C. This is characteristic for dominant diamagnetic material accompanied by minor paramagnetic phase. Hysteresis loop and DC demagnetization curve of sample B2-2 suggest

tiny amount of magnetite present in the rock. The saturation magnetisation value of ferrimagnetic phase (0.0677 A/m) has been determined by subtraction of paramagnetic signal from hysteresis loop. Assuming that the ferrimagnetic phase is magnetite of saturation magnetisation 93 $Am2kg^{-1}$ and mass susceptibility 578 x 10^{-8} SI/kg the proportion of magnetite in the sample is 2.33 x 10^{-3} ‰ and its contribution to the volume susceptibility is $3.51x10^{-8}$ SI. Thus the influence of detected amount of magnetite on mean magnetic susceptibility is considered to be negligible.

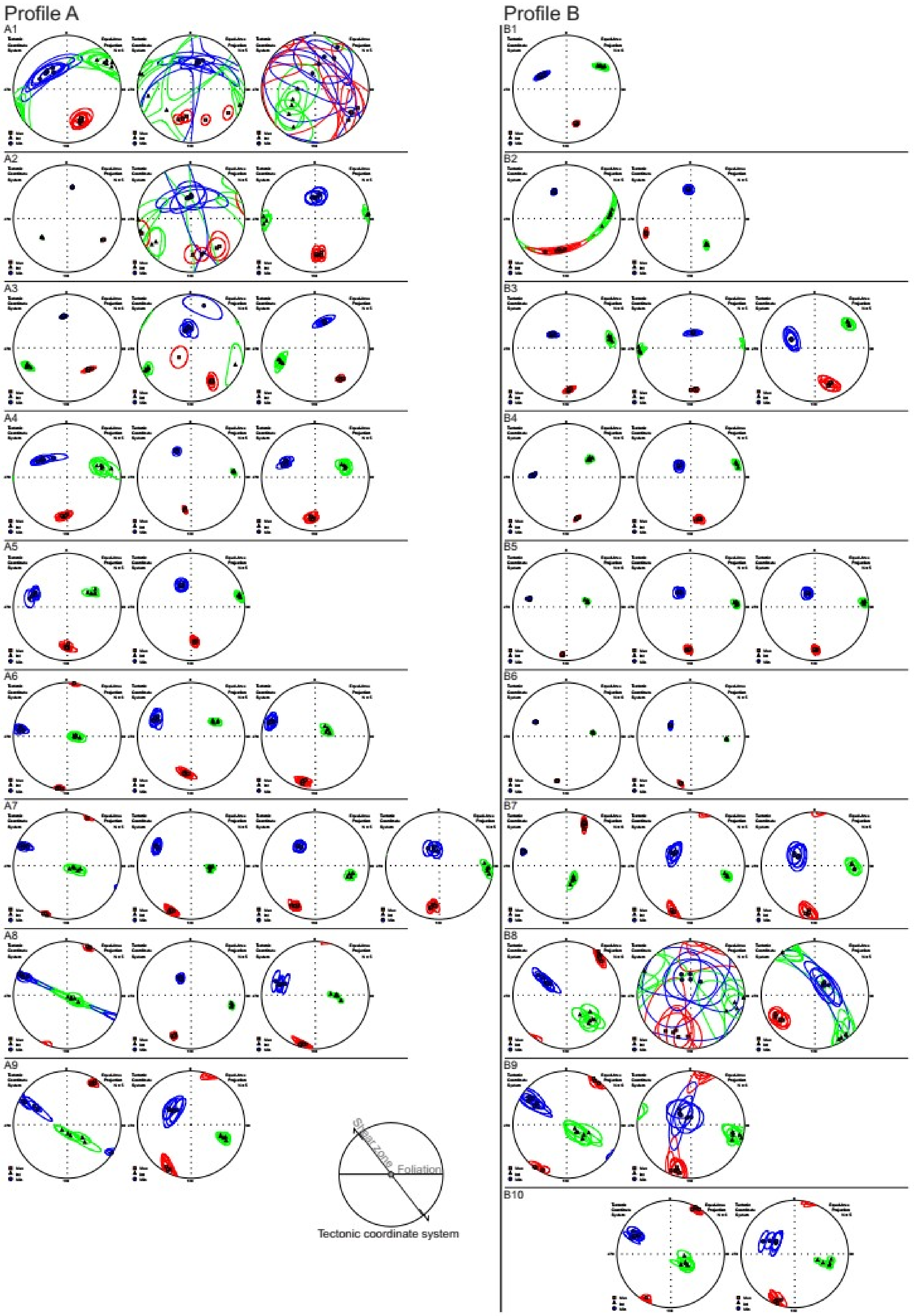

Supplementary Figure S4 | AMS data with confidence ellipses for each measurement. Diagrams are in the same structural framework as in the Supplementary Figure S2.

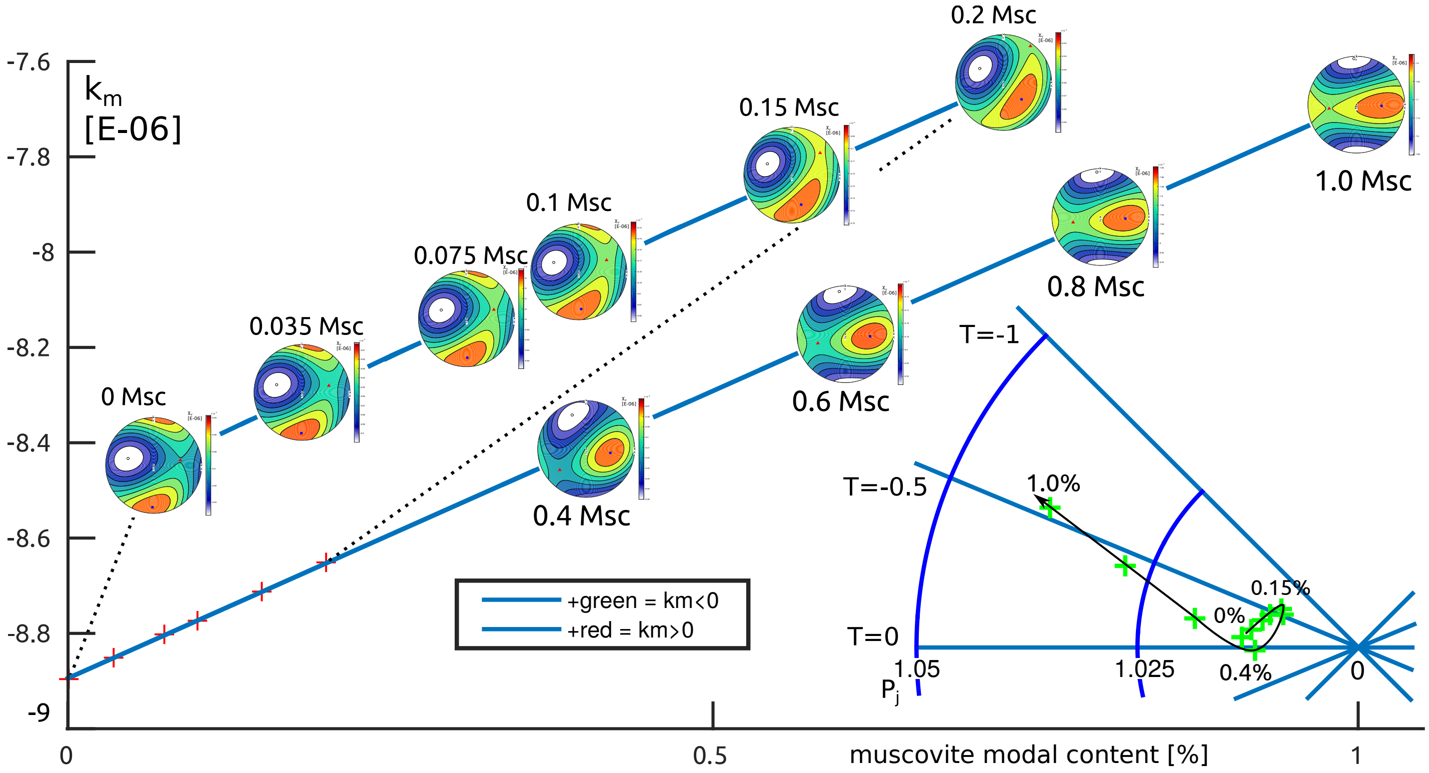

Supplementary Figure S5 | Results of numerical modelling of muscovite contribution to magnetic fabric. Considered third phase (mica) in sample B5, where its orientation is clear with respect to primary fabric (basal plane of mica is within sample's foliation plane). The background for numerical modelling was sample B5 (compare with Fig. 5). Modelled AMS evolution is based on calcite and dolomite single crystal properties (98% calcite and 2% dolomite with 25000ppm Fe) and increasing modal content of Fe-muscovite (0 to 1% muscovite). We also used single grain orientation for mica, as this show the upper boundary of its influence on magnetic properties of sample. We used magnetic tensor for Fe-bearing muscovite deduced from samples Mu303 and Mu401 of similar Fe content to mica in our samples. The AMS tensor for a muscovite single crystal with 2.34 wt.% of FeO is defined with values $k_1=1.21\times10^{-4}$, $k_2=1.18\times10^{-4}$, $k_3=8.71\times10^{-5}$ [SI] and mean $k_m=1.09\times10^{-4}$ [SI][4]. Muscovite is paramagnetic with k_3 susceptibility axis parallel to its crystallographic c-axis and k_1 and k_2 axes within the basal plane.
Graph of k_m vs. muscovite modal content [%] with modelled AMS orientations at specific content levels and P_j - T polar plot of modelled evolution with increasing mica content. AMS diagrams are in the same structural framework as in the Supp. Fig. S2., S4.
Very strong influence of mica on magnetic fabric is observed. Already 0.5% of mica concentration leads to prolate fabric and switch of the k_3 orientation normal to foliation. This is inconsistent with measured magnetic fabric of sample B5 and suggest insignificant contribution of Fe-mica to AMS and therefore we consider our model based on calcite and dolomite as representative with regards to AMS observed for samples affected by the shear zone. Nevertheless, this model can clarify observed obliquity of AMS to primary foliation in samples from outside the SZ, which correspond to principal axes shift for content of mica between 0 and 0.2%. Noteworthy, the model also does not show any significant increase of mean magnetic susceptibility by the mica content limited by magnetic fabric reorientation (0.5%).

[4] Biedermann, A. R., Koch, C. B., Lorenz, W. E. & Hirt, A. M. Low-temperature magnetic anisotropy in micas and chlorite. *Tectonophysics*, **629**, 63–74, DOI: 10.1016/j.tecto.2014.01.015 (2014).
